# Immunohistochemical expression of angiotensin‐converting enzyme 2 in superficial and deep maxillofacial tissues: A cross‐sectional study

**DOI:** 10.1002/hsr2.737

**Published:** 2022-07-20

**Authors:** Noor Allawi, Bashar Abdullah

**Affiliations:** ^1^ Department of Oral Diagnosis College of Dentistry/University of Baghdad Baghdad Iraq

**Keywords:** coronavirus, COVID‐19, protein expression, receptors

## Abstract

**Background and Aims:**

The involvement of maxillofacial tissues in SARS‐CoV‐2 infections ranges from mild dysgeusia to life‐threatening tissue necrosis, as seen in SARS‐CoV‐2‐associated mucormycosis. Angiotensin‐converting enzyme 2 (ACE2) which functions as a receptor for SARS‐CoV‐2 was reported in the epithelial surfaces of the oral and nasal cavities; however, a complete understanding of the expression patterns in deep oral and maxillofacial tissues is still lacking.

**Methods:**

The immunohistochemical expression of ACE2 was analyzed in 95 specimens from maxillofacial tissues and 10 specimens of pulmonary alveolar tissue using a semiquantitative immunohistochemical scoring procedure, taking into account all superficial and deep maxillofacial tissue cells. We also explored the associations of age, gender, and anatomical site with expression scores.

**Results:**

ACE2 was detected in keratinized epithelia (57.34%), non‐keratinized epithelia (46.51%), nasal respiratory epithelial cells (73.35%), pulmonary alveolar cells (82.54%), fibroblasts (63.69%), vascular endothelial cells (58.43%), mucous acinar cells (59.88%), serous acinar cells (79.49%), salivary duct cells (86.26%) skeletal muscle fibers (71.01%), neuron support cells (94.25%), and bone marrow cells (72.65%). Age and gender did not affect the expression levels significantly in epithelial cells (*p* = 0.76, and *p* = 0.7 respectively); however, identical cells expressed different protein levels depending on the site from which the specimens were obtained. For example, dorsal tongue epithelia expressed significantly lower ACE2 scores than alveolar epithelia (*p* < 0.001). A positive correlation was found between ACE2 expression in fibroblasts and epithelial cells (*r* = 0.378, *p* = 0.001), and between vascular endothelial and epithelial cells (*r* = 0.395, *p* = 0.001).

**Conclusion:**

ACE2 is expressed by epithelial cells and subepithelial tissues including fibroblasts, vascular endothelia, skeletal muscles, peripheral nerves, and bone marrow. No correlation was detected between ACE2 expression and patient age or sex while the epithelial expression scores were correlated with stromal scores.

## 1 INTRODUCTION

Angiotensin‐converting enzyme 2 (ACE2) is a cell surface metallopeptidase that was discovered back in 2000 by two independent groups. It is a regulator of the renin‐angiotensin system (RAS) that cleaves angiotensin I and angiotensin II to Angiotensin (1–9) and angiotensin (1–7), respectively.^1^, ^2^ In addition to its physiological functions, ACE2 also serves as a functional receptor for severe acute respiratory syndrome coronavirus 2 (SARS‐CoV‐2).^3^

The role of ACE2 in the pathogenesis of severe acute respiratory distress syndrome (SARS) drew the attention of researchers to its expression in human tissues. Quantitative Reverse Transcriptase Polymerase Chain Reaction (QRT‐PCR) revealed that ACE2 mRNA is expressed in 72 different human tissues. Immunohistochemical studies offer the advantage of providing visual information about the spatial distribution of ACE2 on the cell surface.^4^ ACE2 protein expression was reported in the testis, kidney, heart, lung, bronchioles, nasal mucosa, skin, and vascular endothelium.^5^, ^6^ In the oral cavity, ACE2 was detected in epithelial surfaces and in the salivary glands^7^, ^8^, ^9^; however, ACE2 expression in the majority of deeper oral and maxillofacial tissues such as fibrous tissues, blood vessels, vascular smooth muscles, skeletal muscles, nerves, adipose tissue, bone, and bone marrow is still not thoroughly explored, and many previous immunohistochemical studies were limited by relatively small sample sizes.^7^, ^8^, ^9^, ^10^

Recent reports suggested that airborne and fecal‐oral routes, as well as direct contact and fomites are possible means of SARS‐CoV‐2 transmission.^11^, ^12^ The oral and nasal tissues are potential targets for the viral infection with symptoms ranging from mild gustatory or olfactory dysfunction to a life‐threatening tissue necrosis known as SARS‐CoV‐2 associated mucormycosis.^13^, ^14^ The differences in ACE2 expression may be implicated in the severity of infections, and a number of factors such as age, gender, and ethnicity, are believed to affect the expression of ACE2.^15^

Studying the patterns of ACE2 expression in oral and nasal tissues is essential to identify the tissues that could be involved in viral transmission, tissues that are susceptible to damage, and tissues that act as reservoirs for the virus. Identifying the factors that affect ACE2 levels is an essential step in understanding the maxillofacial manifestations of SARS‐CoV‐2 infections, and may help identify individuals who are more susceptible to severe infections and tissue necrosis. To the best of our knowledge, this is the first study that explores ACE2 expression in all major cell types that constitute the oral and maxillofacial tissues, including superficial tissues, deep soft tissues, bone, as well as pulmonary epithelium.

## 2 METHODS

### 2.1 Tissue samples

We utilized the archives of the Laboratory of Oral Pathology of the College of Dentistry/Baghdad University and the Institute of Forensic Medicine in Baghdad to obtain paraffin‐embedded tissue blocks. Specimens were randomly obtained from nine locations that represent the maxillofacial and pulmonary alveolar tissues, namely, keratinized gingival and palatal tissues, non‐keratinized buccal and labial tissues, ventral tongue, lateral tongue, dorsal tongue, major salivary glands, minor salivary glands, nasal cavity, and pulmonary alveoli. Only the non‐pathological biopsy margins were considered for the analysis. Hematoxylin and eosin (H&E) slides were prepared and examined by a qualified pathologist to verify the suitability of each specimen for inclusion in the study. The exclusion criteria included history of systemic disease or smoking/alcohol consumption (as indicated by the attached clinical reports), malignant and benign neoplastic tissues, tissues with inflammatory infiltrates, tissues with focal or total necrosis, and poorly preserved/small specimens. Patient age and sex were retrieved from the attached clinical reports. A total of 95 maxillofacial specimens and 10 pulmonary alveolar specimens met our inclusion criteria. Patients were classified according to age into young (<18 years), middle‐aged (18–49 years), and patients aged (>49 years). The study protocol was approved by the ethical committee of the College of Dentistry/Baghdad University (Reference number 301721).

### 2.2 Immunohistochemistry

ACE2 protein was detected immunohistochemically using the DAB + chromogen visualization method. The primary antibody was supplied by MyBioSource, San Diego, California, USA (Rabbit anti‐human ACE2 (SARS receptor), MBS9212149, 1:100 dilution). Primary antibody binding was detected using EnVision Flex detection kit (code number K8000) (Agilent DAKO).

Four‐micrometer (4 µm) tissue sections were mounted on positively charged slides. Following wax elimination and rehydration, heat‐induced epitope retrieval (HIER) technique was employed to unmask target epitopes. Tissues were washed by phosphate‐buffered solution (PBS) for five minutes before each new step. Blocking endogenous peroxidase activity was achieved by incubating the tissue in a blocking solution consisting of hydrogen peroxide, NaN3, and detergent for 15 min. Nonspecific antigen binding was blocked by incubating the tissue for 1 h at 37°C in a blocking buffer. The tissues were incubated with the primary antibody for 30 min at 37°C. The tissues were then incubated with goat anti‐rabbit immunoglobulins coupled with dextran and peroxidase for 30 min at 37°C. A solution of DAB + chromogen was applied for 5 min, followed by counterstaining with hematoxylin. Tissue sections were then dehydrated, immersed in xylene, and a cover slip with a drop mounting medium was placed over the slides. Normal human kidney was used as a positive control. Negative control slides were prepared by eliminating the primary antibody to exclude the possibility of nonspecific binding.

### 2.3 Scoring procedure

A semiquantitative immunohistochemical scoring procedure was employed. The staining intensity score was calculated as 0 (no staining), 1 (light yellow staining), 2 (brown staining), and 3 (dark brown staining). The positive cell proportion score was calculated as 0 (0%), 1 (<10%), 2 (10%–35%), 3 (35%–70%), and 4 (>70%). Expression scoring was performed by multiplying the staining intensity score by the positive cell proportion score to calculate the staining index (SI). The SI was calculated separately for each cell type, for example, the SI of epithelial cells was calculated independently of that of fibroblasts and vice versa. A score of 4 or less was regarded as weak expression while a score of 6 or more indicated strong expression. The scoring procedure was blindly performed by two qualified investigators, and the SI values were averaged for further comparative evaluation as described previously.^16^

### 2.4 Statistical analysis

For groups in which scores were normally distributed, the two‐tailed *t*‐test was used to compare the means of two groups. One‐way analysis of variance (ANOVA) was used to compare multiple groups. For groups in which scores were not normally distributed, Mann–Whitney *U* and Kruskal–Wallis tests were used instead. The Chi‐square test was used to compare ACE2 expression levels between the different age groups, and between males and females. Pearson's correlation coefficient was used to analyze the relationship between stromal and epithelial ACE2 expression. A *p* value of less than 0.05 was considered statistically significant. All statistical analyses were carried out using IBM SPSS statistics 26 software.

## 3 RESULTS

The mean age of the patients was 38.58 years, 54 patients were males and 51 were females. ACE2 expression in epithelial and stromal cells was not significantly different between males and females (*p* = 0.69). Patients of different age also showed no significant difference in ACE2 expression in epithelial tissues (Pearson's chi‐square test, *p* = 0.76), fibrous tissues (Fisher's exact test *p* = 0.29, 95% confidence interval [CI] = 0.192–0.387), or vascular endothelia (Fisher's exact test *p* = 0.44, 95% CI = 0.325–0.552).

ACE2 staining in positive cells was cytoplasmic, membranous, and nuclear. Information on the patterns of staining in positive cells are presented in Table 1.

**Table 1:** Patterns of ACE2 staining in positive cells

Cell type	Membranous staining	Cytoplasmic staining	Nuclear staining
Squamous epithelial	55.40%	48.02%	44.72%
Nasal respiratory epithelial	66.4	71.5	63.2
Pulmonary alveolar	>99%	>99%	>99%
Fibroblast	>99%	>99%	>99%
Vascular endothelial	>99%	>99%	>99%
Mucous acinar	>99%	0	0
Serous acinar	>99%	59.7	30.5
Skeletal muscle	>99%	72.6	0
Neuron support cells	>99%	>99%	>99%
Adipocyte	>99%	0	0

Abbreviation: ACE2, angiotensin‐converting enzyme 2.

ACE2 was detected in keratinized epithelial cells (57.34%) and non‐keratinized epithelial cells (46.51%). In 57/66 (86.36%) of the specimens that consisted of squamous epithelia, ACE2 expression was more prominent in the basal and suprabasal layers while the superficial layers were stained inconsistently for ACE2. In the subepithelial connective tissue, fibroblasts and vascular endothelial cells expressed positive ACE2 staining.

Epithelial cells expressed different ACE2 scores depending on the location of tissue. The keratinized gingival mucosa and the mucosa of the lateral surface of the tongue expressed ACE2 scores that were not significantly different from those of the pulmonary alveolar epithelium. However, ACE2 staining was lower in lower lip and cheek epithelium (*p* = 0.01), ventral tongue epithelium (*p* = 0.04), and dorsal tongue epithelium (*p* < 0.001). There was no significant difference in ACE2 staining between keratinized epithelia of the gingiva/hard palate and the non‐keratinized epithelia of the lower lip/cheek (*p* > 0.99). The average SI scores of different tissue groups are presented in Table 2.

**Table 2:** ACE2 immunohistochemical expression scores in different tissues presented as mean ± standard deviation

Site^a^	Mean ± SD							
Cell/tissue	Gingiva/hard palate (*n* = 14)	Lip/cheek (*n* = 15)	Dorsal tongue (*n* = 13)	Lateral tongue (*n* = 12)	Ventral tongue (*n* = 13)	Nasal cavity (*n* = 14)	Major salivary gland (*n* = 12)	Minor salivary gland (*n* = 10)^b^
Epithelia	8.5 ± 3.1	6.8 ± 2.6	3.3 ± 2.2	9 ± 1.8	6.9 ± 3.7	9.9 ± 2.8		
Fibroblast	10.4 ± 1.9	8.2 ± 2.7	8.8 ± 2.2	11 ± 1	10.1 ± 2.2	9.3 ± 3.1		
Skeletal muscle				11.2 ± 0.9	10.3 ± 2.3			
Peripheral nerve					12 ± 0.0			
Serous acini							11 ± 1.4	
Mucous acini							9.7 ± 2.4	8.9 ± 3.3
Salivary duct							11.3 ± 1.6	10 ± 2.4
Calcified bone						0		
Bone marrow						10.4 ± 1.9		

^a^ Pulmonary alveolar and vascular endothelial specimens were not mentioned in this table due to the non‐normal distribution of their SI values.

^b^ Minor salivary gland group included some tissues from lower lip/cheek and ventral tongue groups due to the presence of minor salivary glands in these locations.

The nasal respiratory epithelia expressed ACE2 in 73.35% of the cells while pulmonary alveoli expressed ACE2 in (82.54%) of the cells. No significant difference in ACE2 expression was detected between the nasal and pulmonary epithelia (*p* > 0.99).

**Figure 1:**
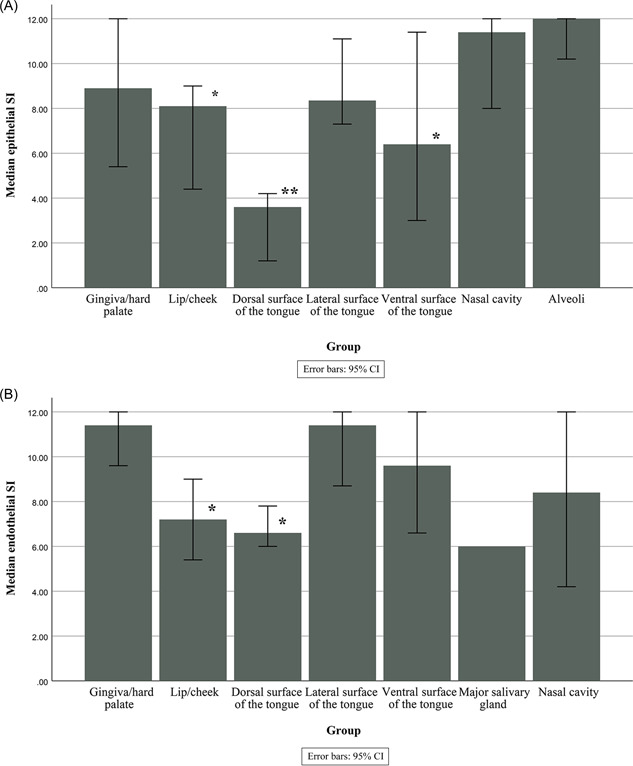
(A) Median epithelial ACE2 expression in different sites. *p < 0.05 when compared to pulmonary epithelia, **p < 0.01 when compared to pulmonary epithelia. (B) Median vascular endothelial expression of ACE2 in different sites. *p < 0.05 when compared to gingiva/hard palate. ACE2, angiotensin‐converting enzyme 2.

ACE2 staining was detected in fibroblasts (63.69%), and it was consistent across different oral and respiratory tissues. Vascular endothelia expressed ACE2 in 58.43%, of the cells, and the expression was lower in the blood vessels below the dorsal epithelium of the tongue compared to gingival mucosa (*p* = 0.002). Blood vessels below non‐keratinized epithelium also expressed lower endothelial ACE2 scores than those from the keratinized gingiva (*p* = 0.01). The median epithelial and endothelial ACE2 expression scores are presented in Figure 1.

In salivary glands, ACE2 was expressed in mucous acinar cells (59.88%), serous acinar cells (79.49%), and salivary duct cells (86.26%). There was no significant difference in ACE2 expression between major and minor salivary glands (*p* = 0.59). ACE2 expression was not significantly different between serous and mucous acini (*p* = 0.4); however, in mucous acini, the expression was mostly limited to the basal parts of the cell membrane while serous acini expressed ACE2 on the cell membrane, in the cytoplasm, cytoplasmic granules, and nuclei.

**Figure 2:**
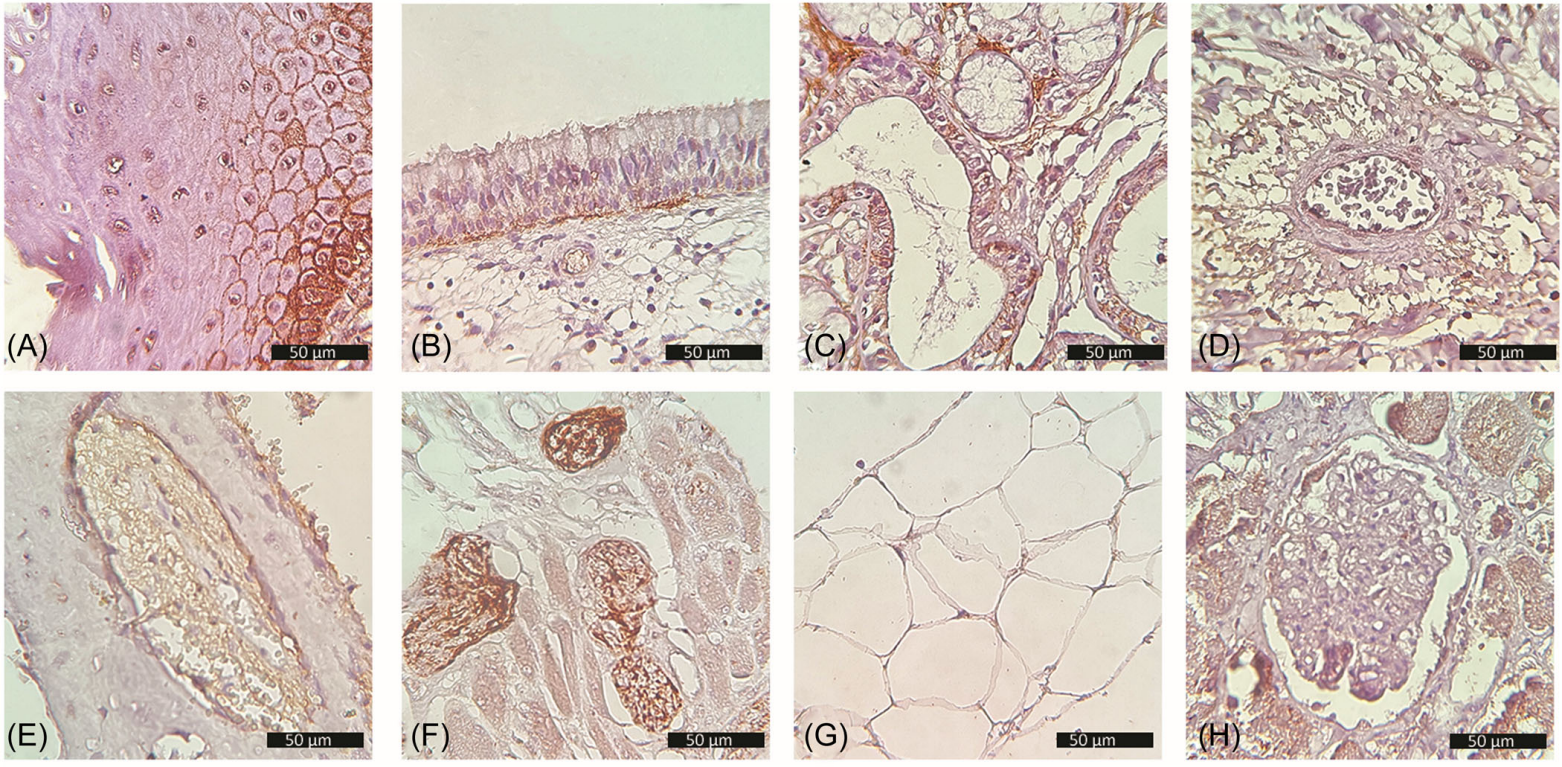
Immunohistochemical expression of ACE2. (A) Oral mucosa showing strong membranous and cytoplasmic expression in the basal layer, membranous expression in suprabasal layers, and negative expression in superficial layers. (B) Respiratory nasal epithelium showing expression in the basal layer. (C) Expression in salivary ducts and acini. (D) Positive expression in vascular endothelium but not the vascular smooth muscles. (E) Positive expression in bone marrow but not in bone (nasal cavity). (F) Strong expression in neuron support cells, skeletal muscle fibers show weaker expression while neurons show no expression (tongue). (G) Membranous expression in adipocytes (parotid gland). (H) Positive control tissue (kidney). ACE2, angiotensin‐converting enzyme 2.

Skeletal muscle fibers from the tongue tissue groups, and adipocytes from buccal and parotid tissues stained positively for ACE2, (71.01%), and (46.51%), respectively. In peripheral nerves of the tongue, the neuron support cells yielded a very strong ACE2 signal in 94.25% of the cells; however, the neurons were consistently negative. In all tissue groups, less than 20% of inflammatory cells, such as lymphocytes, plasma cells, neutrophils, and macrophages, expressed positive ACE2 signals while the vascular smooth muscles, and red blood cells were consistently negative. In the nasal cavity group, bone trabeculae lacked ACE2 staining while the bone marrow cells stained positively in 72.65% of the cells. Figure 2 shows ACE2 staining in different types of tissues.

**Figure 3:**
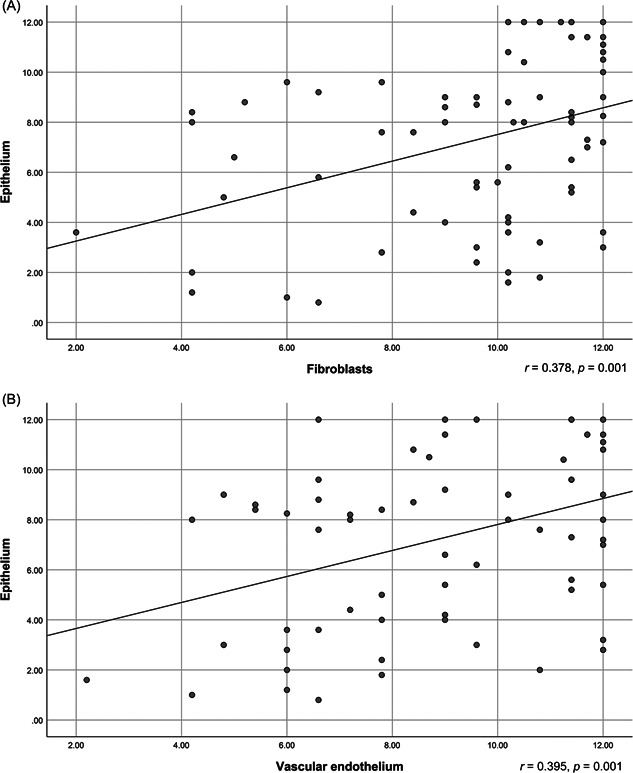
Scatter diagram showing epithelial ACE2 expression against fibroblast expression (A), and vascular endothelial expression (B). ACE2, angiotensin‐converting enzyme 2.

Remarkably, ACE2 expression in oral and nasal epithelial cells was found to be positively correlated with ACE2 expression in the underlying connective tissue stroma. This relationship is demonstrated as a straight upward sloping line in Figure 3.

## 4 DISCUSSION

Since the nasal and oral cavities are both implicated in SARS‐CoV‐2 infections, this study was carried out to provide a comprehensive understanding of ACE2 expression giving consideration to the wide variety of cells that constitute the maxillofacial tissues. We also sought to identify the factors that affect ACE2 levels in maxillofacial and pulmonary alveolar tissues.

In this study, the relationship between ACE2 expression and patient age and gender was explored. Although ACE2 SI values did show interindividual variation, there were no statistically significant differences between males and females or between younger and older patients. Some studies contradict this result. A review by Getachew and Tizabi in 2021 suggested that ACE2 declines with age, especially in males.^15^ One immunohistochemical study reported higher ACE2 levels in the oral epithelial cells of patients older than 49 years.^10^ Multiple studies, however, agree that males do not express significantly different ACE2 levels compared with females, and that ACE2 expression does not seem to be correlated with the patient's age.^16^, ^17^, ^18^, ^19^

This study agrees with a previous immunohistochemical and RNA sequencing study that reported high ACE2 expression in respiratory epithelia.^20^ However, One RNA sequencing study contradicted this result suggesting that the expression level in the respiratory epithelium was low, and that olfactory epithelium was comparatively more markedly positive for ACE2.^21^ However, RNA studies usually have the limitation that they cannot measure the protein contents of the tissue directly.^4^

One noticeable feature of ACE2 expression was that identical cells expressed different ACE2 levels depending on the sites from which the tissue specimens were obtained. For example, the keratinized mucosa of the dorsal surface of the tongue expressed lower ACE2 levels than the keratinized mucosa of the gingiva. Previous studies that performed public bulk RNA sequencing data set analysis,^22^ single‐cell sequence data set analysis, and immunohistochemistry^9^ detected different epithelial ACE2 levels in different oral sites; however, they reported that ACE2 levels in the tongue were higher than in other oral sites.^9^, ^22^ The fact that these studies did not subdivide the tongue mucosa into dorsal, lateral, and ventral may explain this contradiction. The SI scores of endothelial cells varied according to the locations from which the samples were obtained. The variability in endothelial ACE2 expression was noted by An et al. in 2021. In their immunohistochemical study, capillary endothelial cells from pancreatic acinar tissues were almost all positive while capillary endothelial cells from the liver, stomach, and colon were all negative for ACE2 staining. Another immunohistochemical study reported positive endothelial ACE2 staining in the heart, endocrine glands, and pancreas, while vascular endothelial cells in the kidney, liver, testis, stomach, small intestine, and colon were negative for ACE2. It is likely that ACE2 performs other physiological functions besides its role in the renin‐angiotensin system.^7^ This might explain the variability in its levels in different organs.

Some reports implicate SARS‐CoV‐2 infection in causing oral manifestations such as dysgeusia, salivary dysfunction, and oral mucosal lesions.^13^ The high ACE2 signals detected in our study are in agreement with previous studies,^9^, ^22^ and may explain why some patients develop these symptoms. In 2020, Sakaguchi et al. noticed that ACE2 expression was more pronounced in the basal and spinous layers compared with superficial layers. In our study, the same observation was noted. This expression pattern may explain why oral epithelia are usually spared during infection. In respiratory and gastrointestinal epithelia, ACE2 was abundantly expressed on the luminal side rather than the basal side of the epithelium.^17^, ^23^ This might explain why respiratory and gastrointestinal symptoms are usually more pronounced than oral symptoms. It may be tempting to speculate that patients who develop more severe oral manifestations of SARS‐CoV‐2 infections express relatively high ACE2 levels in the superficial layers of oral epithelium; however, further studies are required to test this hypothesis. One study reported that desquamated epithelial cells are candidate sites for SARS‐CoV‐2 replication,^24^ and that aspiration of such cells may contribute to lower respiratory tract infections. If true, this would further complicate the course of the infection in patients with higher than average ACE2 levels in superficial epithelial cells.

Despite the high levels of ACE2 reported in oral tissues,^9^, ^22^ the oral involvement is usually less serious than in the respiratory and the lower gastrointestinal tissues, and oral lesions resolve completely within 14 days.^25^, ^26^ The oral cavity consists of innate and adaptive immune mechanisms,^27^ expresses low levels of ACE2 in the superficial layers of the epithelium,^7^ and is accessible to manual cleansing. Proper oral hygiene was suggested to minimize the risk of pneumonia.^27^

The detection of high levels of ACE2 in the salivary ducts and acinar cells is in agreement with previous immunostaining studies,^7^, ^8^ and supports the conjecture that salivary glands may act as reservoirs for the virus.^28^ The expression was consistent in major and minor salivary glands, and both serous and mucous acini expressed comparable levels of ACE2. One immunohistochemical study detected ACE2 in salivary ducts but not in salivary acini.^29^ The small sample size in their study and the fact that they did not use HIER technique may be the reason behind this discrepancy.

Recently, SARS‐CoV‐2 infections have been linked to a sudden rise in the number of mucormycosis infections and deep necrosis of the maxillofacial tissues, and uncontrolled diabetes mellitus and corticosteroid treatment were reported to be the major risk factors of these infections.^30^ Although the fungal organisms were recognized as the main cause of tissue damage, the role of SARS‐CoV‐2 has not yet been excluded. The high levels of ACE2 in vascular endothelial cells and deep maxillofacial tissues may be implicated in tissue destruction. Tissue destruction could be either a direct consequence of the combined viral and fungal infection, or it could be an indirect consequence of the effects of ACE2 on the immune response by causing a cytokine storm.^15^ It has been reported that Oral SARS‐CoV‐2 infections are associated with superinfections with other oral pathogens; therefore, the destruction of epithelial and stromal tissues may be precipitated by the combined actions of SARS‐CoV‐2 and other pathogens such as mucor fungi.^27^

One remarkable finding of this study was that epithelial ACE2 expression scores were positively correlated with stromal ACE2 scores. This was also true for samples from the nasal cavity. To the best of our knowledge, this finding was not reported previously in the literature; therefore, factors that are implicated in this epithelial‐stromal correlation are not yet understood. ACE2 expression is believed to be affected by local and systemic factors such as smoking, salt, and female hormones that may exert their effects on both epithelial and connective tissue cells simultaneously.^7^ Additionally, It was reported that genetic factors affect ACE2 expression, possibly resulting in higher or lower expression levels in multiple tissues including both epithelial and stromal tissues.^31^ ACE2 is believed to perform functions besides its role in the renin‐angiotensin system such as inflammatory response regulation,^15^ and taste perception,^7^ and further studies are required to explore the possibility ACE2 upregulation/downregulation according to the functions it performs in various organs. A soluble form of ACE2 has been identified.^32^ It is possible that soluble ACE2 may be secreted by one cell and taken up by other cells in the vicinity resulting in an increased expression in both epithelial and subepithelial cells. However, investigating whether or not the features of ACE2 mentioned previously are truly implicated in the epithelial‐stromal expression correlation is beyond the scope of this study, and future studies dedicated to investigating this observation are required.

## 5 CONCLUSIONS

ACE2 expression was detected in epithelial cells and subepithelial tissues including fibroblasts, vascular endothelia, skeletal muscles, peripheral nerves, and bone marrow. No correlation was detected between ACE2 expression and patient age or sex while the epithelial expression scores were correlated with stromal scores.

## AUTHOR CONTRIBUTIONS

**Noor Allawi**: Conceptualization; data curation; formal analysis; investigation; methodology; resources; validation; visualization; writing–original draft; writing–review and editing. **Bashar Abdullah**: Conceptualization; data curation; investigation; methodology; resources; validation; visualization; writing–review and editing.

## CONFLICT OF INTEREST

The authors declare no conflict of interest.

## ETHICS STATEMENT

This study was approved by the ethical committee of the College of Dentistry at the University of Baghdad (Reference number 301721).

## TRANSPARENCY STATEMENT

Noor Allawi affirms that this manuscript is an honest, accurate, and transparent account of the study being reported; that no important aspects of the study have been omitted; and that any discrepancies from the study as planned (and, if relevant, registered) have been explained.

## Data Availability

The data that support the findings of this study are available from the corresponding author upon reasonable request.
